# Sociodemographic Factors Associated with Women's Perspectives on Male Involvement in Antenatal Care, Labour, and Childbirth

**DOI:** 10.1155/2020/6421617

**Published:** 2020-01-25

**Authors:** Shamsudeen Mohammed, Ibrahim Yakubu, Issahaku Awal

**Affiliations:** ^1^Postgraduate Programme, Department of Population, Family and Reproductive Health, School of Public Health Kwame, Kwame Nkrumah University of Science and Technology, Kumasi, Ghana; ^2^Department of Nursing, Ghana College of Nurses and Midwives, Nalerigu, Ghana; ^3^Department of Health Education and Promotion, Tehran University of Medical Sciences, Tehran, Iran; ^4^Department of Nursing, Nursing and Midwifery Training College, Gushegu, Ghana; ^5^Department of Medicine, Renal Dialysis, Tamale Teaching Hospital, Tamale, Ghana

## Abstract

**Background:**

Evidence suggests that in patriarchal societies such as Ghana, access to and survival of maternal and child healthcare services require the active involvement of men. However, interventions to promote men's involvement in maternal and child health care are less likely to succeed if the views and concerns of women are not considered. This study provides an understanding of women's perspective on men's involvement in antenatal care, labour, and childbirth in the Northern Region of Ghana.

**Methods:**

Data for this cross‐sectional study were collected from 300 pregnant women using a structured questionnaire. Logistic regression models were then used to determine the socio‐demographic factors associated with women's perspectives on men's involvement in antenatal care, labour, and childbirth.

**Results:**

The mean age of the participants was 28 (SD = 5.21) years. More than four-fifths of the women in this study express the desire for male partner involment in natenatal care (ANC) services (*n* = 258, 86%) and as companions during labour and child birth (*n* = 254, 84.7%). We found that married women were 9.8 times more likely (95%CI 1.59, 60.81) to encourage male involvement in ANC compared to women who were unmarried. The probability of encouraging male involvement in ANC decreased with increased level of education among the women while support for male companionship during childbirth increased significantly with an increased level of education. After accounting for the effect of other significant covariates, there was good evidence to suggest that married women (*p* = 0.002), women with only primary/Junior High School education (*p* = 0.048) and those with two (*p* = 0.010), three (*p* =  0.008), or ≥4 (*p* = 0.044) previous pregnancies had a desire for male partner involvement in ANC while women who attained secondary (*p* = 0.004) or tertiary (*p* = 0.001) level education expressed the desire for male companionship in labour and childbirth in the adjusted model.

**Conclusion:**

Male involvement in antenatal care, labour, and childbirth received overwhelming support from the women in this study.

## 1. Background

A woman's probability of death due to pregnancy is unacceptably high in low and middle‐income countries than in high‐income countries [1]. It is estimated that nearly 30 million women in Africa become pregnant each year and about 250,000 of them die from pregnancy and childbirth‐related causes [2] even though most pregnancy and childbirth complications can be prevented or treated by skilled care during pregnancy, childbirth, and the immediate postnatal period [1]. Furthermore, antenatal care (ANC) reduces maternal and perinatal morbidity and mortality both directly, through early detection and treatment of pregnancy‐related complications, and indirectly, through the identification of women and girls at increased risk of developing complications during labour and delivery [3]. For instance, antenatal care decreases the likelihood of maternal anaemia during pregnancy and the delivery of a premature or low birth weight baby [3, 4]. Notwithstanding the crucial role of antenatal care in maternal and new‐born health, only 40% of all pregnant women in low‐income countries had the recommended antenatal care visits in 2015 [1].

Globally, several studies and reports have highlighted the positive influence male involvement has on the successful implementation of maternal and child health programmes and interventions. For instance, available evidence suggests that in patriarchal societies such as Ghana, access to and survival of maternal and child healthcare services requires the active involvement of men [5, 6]. Men in patriarchal settings have tremendous control over their spouses and a woman must receive permission and money from her husband to seek healthcare [7–9]. As decision‐makers for families, men in some parts of Northern Ghana consult with soothsayers to decide treatment for pregnant women and serve as the final authority on where and when pregnant women should seek medical care [10]. This is regardless of the fact that for some obstetric complications such as haemorrhage, the window of opportunity to respond and save the life of the mother may be measured in hours [2].

Despite the crucial role of men in maternal and child health, it is extremely unlikely to find a husband at an antenatal clinic or in a delivery room in many low and middle‐income countries. In a study conducted in Johannesburg, only 14% of women reported that a partner had attended ANC with them during their current pregnancy [11]. Similarly, a study among married men in Nepal reported 39.3% of male involvement in ANC [8]. Men understand antenatal care as an affair for women and thus inappropriate for them. In some communities, men who rise above these strict gender roles to support their wives during pregnancy and accompany them to the antenatal clinic are ridiculed and stigmatised by other members of their community [10].

Active male participation in antenatal care and childbirth can play an important role in addressing the first and second delays in seeking care: thus delay in recognising problems and deciding to seek care and delay in reaching care [8, 12]. The presence of a male partner in a delivery room will provide emotional support for the mother, decrease pain perception, establish an early relationship between a father and the infant and perhaps encourage the practice of family planning [13]. Notwithstanding the benefits of involving men in maternal and child healthcare, it is unclear how women view such new developments in Sub‐Saharan Africa [6]. In addition, many studies in the area focus on self‐reported behaviours of men on the barriers and determinants of male involvement without seeking the perspectives of women on the subject [8, 14, 15]. Interventions to promote men's involvement in maternal and child health care are less likely to succeed if the views and concerns of women are not considered [6]. This study provides an understanding of women's perspective on men's involvement in antenatal care, labour, and childbirth in the Northern Region of Ghana.

## 2. Materials and Methods

This cross‐sectional study was conducted at the antenatal clinic of Tamale Teaching Hospital (TTH), a tertiary health facility in the Northern Region of Ghana. The hospital serves as a referral centre for all the primary and secondary health facilities in the three regions of the North and the northern part of Brong‐Ahafo Region. It provides advanced clinical health services, collaborates with the University for Development Studies, Tamale, for the training of undergraduate and postgraduate students, and undertakes research that influences treatment and policy in the health sector [16]. The institutional review committee of the Tamale Teaching Hospital reviewed the study protocol and granted approval for the study. The purpose of the study and the rights of participants during the study were clearly explained to each participant and written consent obtained for their participation. The participants were informed they could refuse to answer any questionnaire they are not comfortable with or withdraw from the study at any point without any condition or threat. Only the authors had access to the information collected from participants and the confidentiality of the information was ensured in accordance with the data protection act.

### 2.1. Study Population, Sample Size, and Sampling Method

Three hundred pregnant women were recruited for the study. In line with the inclusion criteria of the study, only pregnant women who were attending ANC at the Tamale Teaching Hospital were enrolled into the study. Pregnant women who were unwilling to give consent and those who did not attend ANC at TTH were excluded from the study. Cochran's formula was used to estimate the sample size for the study using 14% prevalence of male partner attendance at the antenatal clinic [11], an assumption of 95% confidence interval, and 5% degree of error. A minimum sample size of 185 was estimated. However, the final sample size was increased to 300 to boost the power of the study. Systematic random sampling method was used to recruit pregnant women for the study. In this type of probability sampling method, study participants are chosen at regular intervals (estimated by dividing the projected population size by the sample size) from a sample frame after randomly selecting the first participant [17]. A review of the ANC attendance register for the month prior to the period of data collection showed that an average of 80 pregnant women visits the clinic for ANC services daily. Data collection for the study was scheduled to take place within a month during which nearly 1800 pregnant women were expected to visit the antenatal clinic. The estimated monthly attendance was divided by the sample size (300) to give a sampling interval of 6. The first participant for each day of data collection was randomly selected. After that, every sixth (6^th^) eligible pregnant woman was selected to participate in the study until the sample size was obtained.

### 2.2. Data Collection Procedure

A structured questionnaire was used to collect data from the participants on socio‐demographic characteristics such as age, marital status, religion, ethnicity, educational level, and occupation; male involvement in antenatal care; and male partner companionship during labour and childbirth. For the purposes of this study, “male involvement” was defined as the attendance and participation of men in antenatal care services during the visits of their spouse and their presence and support during childbirth. The items for the questionnaire were designed after a thorough literature review of similar studies in peer‐reviewed journals [11, 13, 18–20]. A senior midwife and a public health specialist reviewed the questionnaire and deemed the items appropriate and content valid. Three final year nursing students administered the questionnaire to participants. They were trained on how to obtain consent and administer the study questionnaire to the participants. Women visiting the antenatal clinic were informed about the study during health talk at the clinic and assessed for eligibility afterwards. Voluntary consent for participation was sought from eligible participants and the study questionnaire was only administered to women who agreed to participate in the study after the health talk. The research assistants and the principal investigator explained to the participants how to complete the questionnaire and addressed their concerns. Participants who had no formal education and could not read or write were assisted by the research assistants to complete the questionnaire, this was reported in less than 20% of the participants. Prior to data collection, a native speaker proficient in both English language and the main local language (Dagbani) translated the questionnaire into Dagbani. A second native speaker reviewed the translated instrument to ensure clarity and to eliminate ambiguities. The data collectors, who were native speakers of the main local language, then used the translated questionnaire for all participants with no formal education. The principal investigator supervised the data collection exercise and reviewed all completed questionnaires at the end of each day.

### 2.3. Data Analysis

Data were cross‐checked for completeness, coded in Microsoft Excel spreadsheet, and analysed using STATA Version 14.0 (College Station, Texas 77845, USA). Socio‐demographic characteristics and women's perspectives on men's involvement in antenatal care, labour, and childbirth were described in tables using frequencies and percentages. Univariate and multivariable logistic regression models were used to determine socio‐demographic factors associated with women's perspectives on men's involvement in antenatal care, labour, and childbirth estimating Odds ratio with 95% confidence intervals and *p* values. The univariate logistic regression was applied in the initial analysis and factors with *p*‐value < .05 were selected for inclusion in the multivariable logistic regression analysis to determine independent predictors of women's perspectives on men's involvement in antenatal care, labour, and childbirth. In both the Univariate and multivariable regression models, the significance level was set at <.05.

## 3. Results

Table 1 shows the socio‐demographic characteristics of the study sample. More than half (55.3%) of the 300 pregnant women were within the age group of 20 to 29 years with an overall mean age of 28 (SD = 5.21) years. Majority of the women were married (98.3%), Muslims (68.3%), and Mole‐Dagombas (61.0%). A little over two‐fifths (40.3%) of the participants attended or completed secondary education and 50.3% of them were employed in the formal sector. Most (31.7%) of the women were carrying their second pregnancy and only 16.3% of them had had four or more pregnancies. Vaginal delivery (75.2%) was the major mode of delivery among the pregnant women who had given birth in the past.

### 3.1. Women's Perspective on Male Involvement in Antenatal Care

The perspectives of the pregnant women on men's involvement in antenatal care are presented in Table 2. More than four‐fifths (86.0%) of the participants indicated their desire for male involvement in antenatal care services. Most of them said men would learn about pregnancy and childbirth (33.9%); and how to support women during pregnancy (32.5%) when they participate in antenatal care. Among the women who did not support male participation in antenatal care, 45.2% believed men do not have time to attend ANC while 22.6% believed pregnancy is women's affair. More than half (56.3%) of the study sample indicated their partners ever accompanied them to the hospital for antenatal care. However, only 33.7% of them said their partners participated in antenatal care services. Among the women that said their partners had never accompanied them to the antenatal clinic, 87.0% indicated they had never asked their partners to accompany them to the clinic and 43.9% said they had never asked their partners because they believed they do not have time to attend ANC services.

### 3.2. Association of Women's Socio‐Demographic Factors and Male Involvement in ANC

Table 3 presents the association of women's socio‐demographic characteristics and men's involvement in ANC. The majority of women in the age range of 20–29 reported their husbands ever accompanied them to ANC (62.7%). However, only 38.5% of the male partners actually participated in at least one ANC service. In the case of pregnant women who were less than 20 years, none of their partners participated in any ANC service whereas for women aged 40–48 years only one participant indicated her partner ever participated in one of the ANC services. More than half of the women who were married (*n* = 167, 56.6%) and those who were Christians (*n* = 70, 73.7%) indicated their husbands accompanied them to ANC; however, most of these male partners did not participate in ANC services. The results in Table 3 clearly revealed that most of the male partners who accompanied their partners to the ANC did not participate in any of the ANC services. There was no significant difference in women's previous mode of delivery and their report of male attendance and participation in ANC.

### 3.3. Women's Perspective about Male Partner Companionship during Labour and Childbirth

Table 4 shows the perspectives of women about male partner presence during childbirth in the delivery room. More than four‐fifths (84.7%) of the women expressed their desire for male partner companionship during labour and childbirth. Of these women, 37.5% believed the presence of a male partner will provide emotional support, 21.5% said it will provide the woman with an opportunity to express her problems to a familiar person and 18.9% indicated men will treat women better if allowed in the delivery room during the delivery of their wives. Most of the forty‐six women who did not support the presence of male partners in the delivery room stated that delivery is a women's affair (38.02%) and that men do not have any role to play in the delivery room (28.10%).

### 3.4. Socio‐Demographic Determinants of Women's Perspective about Male Partner Involvement in ANC

Table 5 presents the odds ratio and 95% confidence intervals of the socio‐demographic determinants of women's perspective about male involvement in ANC. As shown in the unadjusted model, women aged 20–29 years (OR 2.34, 95%CI 0.25, 22.26) and 30–39 years (OR 1.06 95%CI 0.11, 9.93) were more likely to encourage male involvement in ANC when compared with women less than 20 years. However, those aged 40–48 years (OR 0.92, 95%CI 0.07, 11.58) were less likely to encourage male involvement in ANC. Married women were (OR 9.85, 95%CI 1.59, 60.81) more likely to encourage male involvement in ANC compared to women who were unmarried.

We observed that the probability of encouraging male involvement in ANC decreased with increased level of education of women: primary/JHS (OR 3.60, 95%CI 1.08, 12.02) secondary (OR 3.19, 95%CI 1.34, 7.61), and tertiary (OR 1.92, 95%CI 0.81, 4.56). A similar pattern was observed in the adjusted analysis: primary/JHS (OR 3.66, 95%CI 1.01, 13.27), secondary (OR 2.16, 95%CI 0.81, 5.75), and tertiary (OR 1.50, 95%CI 0.58, 3.89).

A significant relationship was observed between the number of pregnancies [gravida 2, *p* = 0.021; gravida 3, *p* = 0.010; gravida 4 or more, *p* = 0.046] and a woman's likelihood to encourage male involvement in ANC. After accounting for the effect of other significant covariates, there was good evidence to suggest that married women (*p* = 0.002), women with only primary/Junior High School education (*p* = 0.048) and those with two (*p* =  0.010), three (*p* = 0.008), or ≥4 (*p* = 0.044) previous pregnancies had a desire for male partner involvement in ANC.

### 3.5. Socio‐Demographic Determinants of Women's Perspective about Male Partner Companionship during Labour and Childbirth

Table 6 presents the odds ratios, confidence intervals, and *p* values of the socio‐demographic predictors of women's perspective about male partner companionship in labour and childbirth. There was no significant relationship between the age of the women, number of pregnancies, marital status of the women, and their perspective about male partner companionship in labour and childbirth. We observed that the odds of a woman encouraging the attendance of men at childbirth increased significantly with increased level of education in both the unadjusted and adjusted models: primary/JHS (OR 2.25, 95%CI 0.89, 5.71); AOR 1.94, 95%CI 0.68, 5.57), secondary (OR 5.11, 95%CI 2.23, 11.72; AOR 4.91, 95%CI 1.69, 14.27), and tertiary (OR 6.19, 95%CI 2.36, 16.25; AOR 9.75, 95%CI 2.51, 37.91). The results revealed that women employed in the formal sector (OR 1.12, 95%CI 0.38, 3.33) were more likely to support the presence of a male companion at delivery. As shown in Table 6, women who were carrying their second pregnancy had 1.76 (95%CI 0.72, 4.29) increased odds of encouraging the presence of a male partner at birth compared to women who were pregnant for the firsts time. However, women who were pregnant for the third time (OR 0.63, 95%CI 0.28, 1.41) and those who were carrying their fourth or more (OR 0.95, 95%CI 0.37, 2.44) pregnancy were less likely to encourage the presence of a male partner at birth. Previous caesarean delivery was significantly associated with a 3.67 (95%CI 1.07, 12.60) increased odds of a woman encouraging male partner presence at delivery. This association was no longer apparent after adjustment with other covariates.

## 4. Discussion

The women in this study expressed favourable support for male partner involvement in antenatal care services and as companions during labour and childbirth, which is in line with earlier studies in several low‐income countries [11, 13, 18, 20, 21]. This was probably because most of the women we surveyed believed that men who attend ANC with their partners acquire useful knowledge on how to support their wife's during pregnancy and childbirth. In line with the expectation of the women, a meta‐analysis of 14 studies on the impact of male involvement on maternal health outcome in low and middle‐income countries found that male involvement was significantly associated with reduced odds of postpartum depression and improved utilisation of skilled birth attendance and postnatal care [22]. Likewise, in Ethiopia, Mohammed et al., found that the probability of attending at least one ANC visit, first ANC visit within first trimester, and utilising skilled facility‐based delivery were higher in women with greater male partner involvement [23]. However, contrary to the current findings, an earlier study in Ghana reported that many Ghanaian women do not want their male partners involved beyond the provision of money and transport for maternal and child healthcare services [6]. In this study, women who did not support male participation in antenatal care said men do not have time to attend ANC and that pregnancy is a woman's affair. Both men and women alike in several studies have expressed similar views across Africa [6, 15, 24, 25].

Despite the desire of the current study participants for their partners to attend ANC, only a little over half of them reported that their partners ever accompanied them to ANC, which is consistent with similar studies in Uganda [14,15], and Kenya [5]. Moreover, approximately two‐thirds of the male partners who accompanied their spouses did not participate in any ANC services. This lack of enthusiasm by most men to accompany their partners and participate in ANC services could be cultural because ANC attendance is considered a woman's affair as expressed in several studies [6, 24, 25]. Men may feel discomforted attending a female dominated program and discussing sexual and reproductive health issues with third parties. Fear of societal stigma and unfriendly clinic environment have also been reported as obstacles to male participation in maternal and child health services [10, 26]. In Ghana, Aborigo et al. found that in the Kassena‐Nankana Districts in the Upper East Region, men who accompany their wives to the antenatal clinic are called “kana‐kadona” or “bakana” which means “women's rivals” or “man‐woman” respectively, suggesting that such men exhibit female tendencies [10]. Men are conscious of these cultural barriers in the community and this may explain why most of the women reported that their husbands have never participated in any ANC service and the lack of interest on their part to ask their partners to accompany them for ANC services.

We found that younger women were more likely to encourage male partner involvement in ANC compared to older women. The reason could be that the younger women were likely to experience anxiety and fear during pregnancy than older women who might have experienced pregnancy several times. There was a significant relationship between a woman's marital status and the likelihood to encourage male involvement in ANC. Married women were 9.8 times more likely to support male partner involvement compared to unmarried women. This is probably because out‐of‐wedlock pregnancies are frowned upon and considered a shameful act in many African societies including Ghana, and this may discourage unmarried women from visiting the clinic with the father of their unborn child. Interestingly, the probability of encouraging male involvement among the women in this study decreased with an increased level in education, which agrees well with the finding of a study conducted in Nigeria [25]. However, further evidence, through large studies, is needed to adequately understand why educated women were not in support of male involvement in ANC.

Regarding the presence of men during childbirth, the majority of the women encouraged it because they believed the presence of a companion would provide emotional support, give them an opportunity to express their problems to a familiar person, and inspire men to treat their wives better after observing the delivery process. Several studies in low‐income countries corroborate these findings [13, 21]. However, the current arrangements of most labour wards in Ghana do not make provision for male partners to be with their wives during childbirth. In most clinical settings, pregnant women are admitted to a labour ward without much privacy to prevent male partners from witnessing the delivery of other women. An earlier study in Ghana reported that men are usually sent away from the delivery room during labour and are only called back after delivery and newborn care was completed [27]. Women who did not support male involvement in childbirth indicated that childbirth was a woman's responsibility and that men do not have a role to play in the delivery room.

We found that the odds of a woman encouraging the attendance of male partner at childbirth increased significantly with increased level of education. This result is consistent with those of other studies [19, 25, 28] and suggests that maternal years of formal education have an influence on healthcare utilisation. In line with the findings of Morhason‐Bello et al. [28], the current study revealed that women who were employed in the formal sector were more likely to ask for male companionship during delivery.

## 5. Limitations

Our study has several limitations that must be considered in the application of our findings. Firstly, the study was conducted in an urban city and the findings may not be applicable to women in rural communities. Secondly, we interviewed only women who visited the antenatal clinic of a single hospital and this may limit the generalizability of the findings to pregnant women who visited other facilities and women who did not utilise antenatal care services during pregnancy. Thirdly, the study employed a cross‐sectional study design, making it difficult to establish a causal relationship between the outcome variables and the explanatory variables.

## 6. Conclusion

Male involvement in antenatal care and childbirth received overwhelming support from the women in this study. Their perspectives were that male involvement would expose men to information about pregnancy and childbirth to help support their wives during pregnancy. Furthermore, the women indicated that the presence of a companion during childbirth would provide emotional support, give them an opportunity to express their problems to a familiar person, and inspire men to treat their wives better. Women's marital status, educational level, and number of pregnancies were identified as significant determinants of a woman's perception of male participation in ANC. Likewise, the educational level of women was found to be a significant predictor of a woman's perception of male involvement in childbirth. Interventions to encourage male involvement in pregnancy, labour and childbirth should consider the provision of male‐friendly environment at antenatal clinic and facilities to accommodate male companions in labour rooms as desired by the women in this study.

## Figures and Tables

**Table 1:** Background characteristics of participants.

Characteristic	Number	Percent
*Age (in years)*		
<20	5	1.67
20–29	166	55.33
30–39	115	38.33
40–48	14	4.67
Mean (SD)	28 (5.21)	

*Marital status*		
Unmarried^a^	5	1.67
Married	295	98.33

*Religion*		
Christianity	95	31.67
Islam	205	68.33

*Ethnicity*		
Mole‐Dagombas	183	61.00
Akan	19	6.33
Gonja	21	7.00
Frafra	18	6.00
Chokosi	11	3.67
Dagati	14	4.67
Others	34	11.33

*Education level*		
No formal education	50	16.67
Primary/JHS	45	15.00
Secondary	121	40.33
Tertiary	84	28.00

*Employment status*		
Unemployed	93	31.00
Formal employment^b^	151	50.33
Informal employment^c^	56	18.67

*Number of pregnancies*		
One	90	30.00
Two	95	31.67
Three	66	22.00
Four or more	49	16.33

*Previous mode of delivery ( n* = 210* )*		
Vaginal birth	158	75.24
Caesarean delivery	52	24.76

^a^Unmarried: single, divorced, widowed, cohabiting. ^b^Formal employment: public and private sector employees. ^c^Informal employment: farmer, artisan, trader.

**Table 2:** Women's perspective on male involvement in antenatal care.

Characteristic	Number	Percent
*Should men participate in ANC services*		
Yes	258	86.00
No	42	14.00

*Why should men participate in ANC services (n = 258)^a^*		
In case of infection, we can both be treated	86	14.1
He will learn about pregnancy and childbirth	207	33.93
He will learn how to support a pregnant woman	198	32.46
It is a demonstration of his love	48	7.87
It will make women feel supported	71	11.64

*Why should men stay away from ANC (n = 42)^a^*		
Many men do not have time	21	45.65
Men need to concentrate on their work	5	10.87
ANC is not a place for a man	5	10.87
Pregnancy is women's affair	15	32.61

*Has your partner ever accompanied you to ANC*		
Yes	169	56.33
No	131	43.67

*Did he participate in any ANC service (n = 169)*		
Yes	57	33.73
No	112	66.27

*Did you ever asked your partner to accompany you to the ANC (n = 131)*		
Yes	17	12.98
No	114	87.02

*Why have you never asked him to accompany you to the ANC (n = 114)^a^*		
I know he will not accept to accompany me	40	16.88
He does not have time to attend ANC with me	104	43.88
He is not always around	57	24.05
He has nothing to do at the clinic	3	1.27
His presence will make me uncomfortable	4	1.69
ANC is for only women	11	4.64
His other wives will be unhappy	18	7.59

^a^Multiple responses were allowed.

**Table 3:** Association of women's socio‐demographic factors and male partner involvement in ANC.

Characteristics	Has your partner ever accompanied you to ANC		*p* value	Did he participate in any ANC service (*n* = 169)		*p* value
	Yes	No		Yes	No	
*Age (in years)*						
<20	3(60.00)	2(40.00)	0.009^a^	0(0.00)	3(100.00)	0.275^a^
20–29	104(62.65)	62(37.35)		40(38.46)	64(61.54)	
30–39	59(51.30)	56(48.70)		16(27.12)	43(72.88)	
40–48	3(21.43)	11(78.57)		1(33.33)	2(66.67)	

*Marital status*						
Unmarried	2(40.00)	3(60.00)	0.656^a^	0(0.00)	2(100.00)	0.550^a^
Married	167(56.61)	128(43.39)		57(34.13)	110(65.87)	

*Religion*						
Christianity	70(73.68)	25(26.32)	<0.001	27(38.57)	43(61.43)	0.263
Islam	99(48.29)	106(51.71)		30(30.30)	69(69.70)	

*Education level*						
No formal education	15(30.00)	35(70.00)	<0.001	0(0.00)	15(100.00)	0.005^a^
Primary/JHS	10(22.22)	35(77.78)		5(50.00)	5(50.00)	
Secondary	81(66.94)	40(33.06)		26(32.10)	55(67.90)	
Tertiary	63(75.00)	21(25.00)		26(41.27)	37(58.73)	

*Employment status*						
Unemployed	56(37.09)	95(62.91)	<0.001	30(44.12)	38(55.88)	0.044
Formal employment	68(73.12)	25(26.88)		17(30.36)	39(69.64)	
Informal employment	45(80.36)	11(19.64)		10(22.22)	35(77.78)	

*Number of pregnancies*						
One	54(60.00)	36(40.00)	<0.001	21(38.89)	33(61.11)	0.510^a^
Two	66(69.47)	29(30.53)		23(34.85)	43(65.15)	
Three	33(50.00)	33(50.00)		10(30.30)	23(69.70)	
Four or more	16(32.65)	33(67.35)		3(18.75)	13(81.25)	

*Previous mode of delivery*						
Vaginal birth	83(52.53)	75(47.47)	0.258	26(31.33)	57(68.67)	0.994
Caesarean delivery	32(61.54)	20(38.46)		10(31.25)	22(68.75)	

^a^Fisher's exact test.

**Table 4:** Women's perspective on male partner companionship during labour and childbirth.

Characteristic	Number	Percent
*Should a man be allowed in the delivery room during the delivery of his wife*		
Yes	254	84.67
No	46	15.33

*Why should men be allowed in the delivery (*n* = 42)^a^*		
To treat women better afterwards	81	18.88
To provide emotional support	161	37.53
It may encourage men to support and practice family planning	33	7.68
Helps the mother bear labour pain	32	7.46
An opportunity for the woman to express her problem to a familiar person	92	21.45
Strengthens the couple's relationship	30	6.99

*Why should men stay away from the delivery room (n = 46)^a^*		
Delivery is women's affair	46	38.02
Men do not have role to play in the delivery room	34	28.1
Men may not love their wives the same way after observing the delivery	18	14.88
He may disturb the health professionals	23	19.01

^a^Multiple responses were allowed.

**Table 5:** Socio‐demographic determinants of women's perspective about male partner involvement in ANC.

Characteristics	Unadjusted model		Adjusted model	
	OR(95%CI)	*p* value	AOR(95%CI)	*p* value
*Age (in years)*				
<20	1.00			
20–29	2.34(0.25, 22.26)	0.458		
30–39	1.06(0.11, 9.93)	0.961		
40–48	0.92(0.07, 11.58)	0.946		

*Marital status*				
Unmarried	1.00		1.00	
Married	9.85(1.59, 60.81)	0.014	37.58(3.74, 377.75)	0.002

*Religion*				
Christianity	1.00			
Islam	1.31(0.66, 2.60)	0.446		

*Education level*				
No formal education	1.00		1.00	
Primary/JHS	3.60(1.08, 12.02)	0.037	3.66(1.01, 13.27)	0.048
Secondary	3.19(1.34, 7.61)	0.009	2.16(0.81, 5.75)	0.123
Tertiary	1.92(0.81, 4.56)	0.140	1.50(0.58, 3.89)	0.400

*Employment status*				
Informal employment	1.00			
Formal employment	0.74(0.26, 2.07)	0.564		
Unemployed	0.67(0.26, 1.74)	0.408		

*Number of pregnancies*				
One	1.00		1.00	
Two	0.29(0.10, 0.83)	0.021	0.19(0.52, 0.67)	0.010
Three	0.24(0.81, 0.71)	0.010	0.17(0.04, 0.63)	0.008
Four or more	0.30(0.09, 0.98)	0.046	0.22(0.05, 0.96)	0.044

*Previous mode of delivery*				
Vaginal birth	1.00			
Caesarean delivery	1.87(0.73, 4.78)	0.190		

**Table 6:** Socio‐demographic determinants of women's perspective about male partner companionship during labour and childbirth.

Characteristics	Unadjusted model		Adjusted model	
	OR(95%CI)	*p* value	AOR(95%CI)	*p* value
*Age (in years)*				
<20	1.00			
20–29	1.93(0.21, 18.22)	0.564		
30–39	1.00(0.11, 9.38)	1.00		
40–48	0.92(0.073, 11.58)	0.946		

*Marital status*				
Unmarried	1.00			
Married	3.80(0.62, 23.42)	0.150		

*Religion*				
Christianity	1.00			
Islam	0.42(0.19, 0.95)	0.036		

*Education Level*				
No formal Education	1.00		1.00	
Primary/JHS	2.25(0.89, 5.71)	0.088	1.94(0.68, 5.57)	0.217
Secondary	5.11(2.23, 11.72)	<0.001	4.91(1.69, 14.27)	0.004
Tertiary	6.19(2.36, 16.25)	<0.001	9.75(2.51, 37.91)	0.001

*Employment status*				
Informal employment	1.00			
Formal employment	1.12(0.38, 3.33)	0.839		
Unemployed	0.46(0.18, 1.18)	0.108		

*Number of pregnancies*				
One	1.00			
Two	1.76(0.72, 4.29)	0.214		
Three	0.63(0.28, 1.41)	0.258		
Four or more	0.95(0.37, 2.44)	0.905		

*Previous mode of delivery*				
Vaginal birth	1.00			
Caesarean delivery	3.67(1.07, 12.60)	0.039		

## Data Availability

The datasets used and/or analysed during the current study are available from the corresponding author on reasonable request.

## Abbreviations

ANC: Antenatal care
TTH: Tamale teaching hospital.
